# The Relationship Between Social Media Use and Disordered Eating in Young Adults Aged 18–30: A Narrative Review

**DOI:** 10.3390/nu17203288

**Published:** 2025-10-20

**Authors:** Danai Athanasoula, Aikaterini Salpa, Fotini Sonia Apergi, Ilias I. Vlachos

**Affiliations:** Department of Psychology, The American College of Greece, GR15342 Athens, Greece; a.salpa@acg.edu (A.S.); tapergi@acg.edu (F.S.A.); ivlachos@acg.edu (I.I.V.)

**Keywords:** social media, disordered eating, review

## Abstract

Background/Objectives: Social media use has become ubiquitous, with governmental bodies and researchers expressing a growing concern about its impact on mental health. This review aims to examine the relationship between social media use and disordered eating in individuals aged 18–30. Specifically, we aim to identify specific patterns of use (including addictive use) that are associated with increased risk for disordered eating. Methods: A search was conducted in March 2025 using PubMed and PsycINFO. Keywords were based on social media platforms and eating behaviors. Inclusion criteria were published studies in peer-reviewed journals from 2015–2025, written in English, with participants aged 18–30, whose disordered eating outcomes were assessed using validated measures. Conclusions: 637 articles were screened, with 28 studies meeting the inclusion criteria. Most studies assessed general social media use, without specifying the platform type. The EAT-26 and EDE-Q scales were used in most research to assess disordered eating. Data were narratively synthesized based on the type of social media variables assessed. Our findings demonstrate a complex relationship between social media use and disordered eating, with more consistent associations being found when the type of content (fitspiration and thinspiration) was the independent variable. These findings align with qualitative findings, which highlighted ambivalence in relation to the effect of social media: it is viewed as both a source of support and social comparison. Avenues for future research include longitudinal studies to understand the interaction between individual factors and social media patterns of use, as well as the utilization of platform-generated data on online engagement patterns.

## 1. Introduction

The effect of social media (SM) on mental health has increasingly gained attention from both researchers and federal governments [1,2]. It is undeniable that these platforms have become ubiquitous; estimates show that there are over 5 billion users, each with an average of seven SM accounts and approximately 143 min spent online daily per user—a figure that is significantly higher for younger generations [3,4,5]. Concerns have been raised, particularly in relation to the rise in image-based content, especially on platforms such as Instagram, TikTok, or Snapchat [1]. This shift, which is mirrored in the user interface of most platforms, has created an online environment characterized by consistent exposure to appearance-related content, engagement in photo-based activities, and consumption of appearance-related information [6]. The appearance-focused environment, beyond spreading beauty ideals, may contribute to their normalization and provide opportunities for endless comparisons [5,7]. Consequently, a growing body of research seeks to understand whether SM are associated with disordered eating (DE), as well as to identify mediating and moderating variables in these associations [1].

Scales such as the Eating Attitudes Test-26 (EAT-26) measure maladaptive and disruptive eating behaviors, thoughts, and emotions, which may or may not reach clinical levels, but are a risk factor for the development of an eating disorder (ED) [7,8]. Indeed, evidence utilizing these scales shows that the risk of eating disorders is on the rise, with research reporting an increase of over 13% for youth, between the years 2013–2021 [9].

Using validated measures such as the Eating Attitudes Test-26, several studies have reported a positive correlation between use of SM and disordered eating, with reviews highlighting the importance of this factor [10,11,12]. Internalization of appearance ideals, upward appearance comparisons, poor body image, and self-objectification are amongst the mediating factors that have been identified in the literature [10,13,14,15,16]. Theoretical frameworks support these findings, as they emphasize that exposure to appearance-focused content facilitates internalization of prevailing beauty ideals, which in turn is associated with greater upward comparison, self-objectification, and poorer body image. These processes are, in turn, linked with maladaptive thoughts, emotions, and behaviors around food [17,18].

More recently, studies assessing significant factors beyond frequency of use have emerged [19]. For example, a recent review concluded that exposure to idealized images and the influencer culture correlates with eating disorder risk [13]. Similarly, previous reviews have found that frequent exposure to, as well as high engagement with, visual content are also associated with increased odds of developing an eating disorder [20,21,22]. However, to our knowledge, few reviews have comprehensively examined different factors, to determine whether specific patterns of SM use are more consistently linked to disordered eating. Furthermore, there is a lack of differentiation between social media addiction and high social media use in the reviews assessed.

This review focused on identifying social media-related risk factors for disordered eating among individuals aged 18–30. This age group was selected due to prior findings that eating disorder risk has increased substantially between 2013–2020 for college populations [23]. Any variables that provide insights in relation to patterns of social media use, as well as disordered eating behaviors, were the primary measures of interest in this review. As a result, social media was not limited to quantitative variables such as duration, frequency of use, and number of platforms used in this study. Variables such as content consumed or types of online behaviors were also considered relevant. Disordered eating in this study refers to both behaviors and attitudes, as measured by validated scales such as the Eating Disorder Examination Questionnaire (EDE-Q) or the EAT-26.

## 2. Materials and Methods

To identify papers for review, a search was performed in March 2025 using the APA PsycNET PsycINFO and PubMed databases. The following search query was used: (“eating disorder symptoms” OR “eating disorder” OR “disordered eating” OR “problematic eating behaviors”) AND (“social media” OR “social networking sites” OR “TikTok” OR “Instagram” OR “Facebook” OR “WeChat” OR “Twitter” OR “X” OR “Snapchat”). No filter was used when conducting the search.

Inclusion criteria were published studies between 2015–2025, in peer-reviewed journals, written in English, with participants aged 18–30, whose social media use and disordered eating were assessed. Our review includes studies that assessed disordered eating using validated scales, as well as measured disordered eating in direct relation to variables associated with social media use. In total, findings from 28 studies were synthesized into a narrative review of the literature. Table 1 outlines key results from these studies.

## 3. Results

The results are displayed below, grouped by type of association examined.Only results relevant to the relationship between social media and disordered eating were examined, including moderating and mediating variables.

### 3.1. Social Media Addiction and Disordered Eating

A small number of studies focused on the association between SM addiction and DE outcomes (N = 3) [7,24,25].

Using the Adult Eating Behaviours Questionnaire, Karam et al. [24] found that individuals addicted to social media were significantly more likely to engage in emotional overeating (adjusted OR according to sociodemographic characteristics: 2.2; 95% CI: 1.42–3.54), score higher in food responsiveness (adjusted OR: 2.13; 95% CI: 1.35–3.36), as well as in hunger (adjusted OR: 2.16; 95% CI: 1.37–3.41). Importantly, significant age differences between the two groups were identified, with individuals classifying as addicted being generally younger [24].

Similarly, using the Dutch Eating Behaviors Questionnaire, significant correlations between excessive social media use, emotional overeating, and restrained eating were found in another study (r = 0.304; r = 0.230, accordingly, all *p* < 0.001). Multiple mediation analyses showed significant indirect paths of excessive time on social media on both restrained eating and emotional overeating through appearance and weight esteem (restrained eating: B = 0.0404, SE = 0.0074 [BCa 95% CI 0.0264–0.0558]; emotional overeating: (B = 0.0231, SE = 0.0056 [BCa 95% CI 0.0137–0.0358]); gender did not moderate the pathway to restrained eating. However, for emotional overeating, appearance esteem mediated this relationship for females but not for males [25].

In one study, after controlling for multiple psychosocial and biological factors, SM addiction was not a significant independent predictor (adjusted odds ratio = 0.48, 95% CI: 0.23–1.01), whereas daily SM use was (adjusted odds ratio = 1.68, 95% CI: 1.05–2.69) [7].

### 3.2. Frequency of SM Use and Disordered Eating

Studies focusing on the frequency of social media use show a complex relationship with disordered eating outcomes.

Five studies report positive associations between frequency of social media use and disordered eating [7,26,27,28,29]. Results from these studies are provided below.

As aforementioned, in a multivariate model that controlled several biological and psychosocial variables—such as body dissatisfaction, desire for a thinner body, and self-esteem—frequency of social media use significantly predicted disordered eating (adjusted odds ratio = 1.68, 95% CI: 1.05–2.69) [7]. Similar findings are reported by Murley et al. [26] (*p* < 0.001) and Christensen-Pacella et al. (2024) (*p* < 0.001), whose models showed that higher social media use predicts disordered eating in young adults [26,27]. In the study by Murley et al. [26], two separate moderated mediation models predicting food and alcohol disturbance (FAD) and disordered eating were also tested. For the FAD model, the indirect effect of social media use on FAD through anxiety was significant at lower (b = 0.21, SE = 0.11, 95% CI [0.01, 0.43]) and average (b = 0.14, SE = 0.06, 95% CI [0.03, 0.27]) levels of social support, but not at higher levels of social support. Similarly, for the disordered eating model, the indirect effect of social media use on disordered eating through anxiety was significant at lower (b = 0.27, SE = 0.11, 95% CI [0.03, 0.49]) and average (b = 0.26, SE = 0.09, 95% CI [0.08, 0.45]) levels of social support, but not at higher levels of social support (b = 0.24, SE = 0.14, 95% CI [−0.03, 0.54]). These findings suggest that the indirect relationships between social media use and both FAD and disordered eating via anxiety can potentially be protected by higher levels of perceived social support [26].

Furthermore, Foster et al. [29] showed that frequency of Snapchat use was associated with drive for thinness (r = 0.24, *p* = 0.02), but not with compensatory eating and drinking behavior (termed ‘drunkorexia’; *p* > 0.05). Although a significant relationship between drunkorexia and snapchat use was not identified, the researchers proceeded with mediation analyses that showed a significant three-path model: higher snapchat use was associated to increased comparisons, which resulted in higher internalization of the thin ideal and drove compensatory drinking and eating behaviors (Mediated Effect [ME] = 0.02, SE = 0.01, 95% CI [0.006, 0.042]). Interestingly, when the path included only appearance comparison, Snapchat use showed a small but significant negative association with DE (ME = −0.01, SE = 0.01, 95% CI [−0.009, −0.003]) [29].

Finally, drive for thinness was found to significantly, albeit weakly, correlate with Facebook usage (r = 0.17, *p* = 0.01) in another study. The authors reported that appearance comparisons on Facebook mediated the link between usage and drive for thinness, with the effect being slightly stronger for comparisons to distant peers (drive for thinness: B = 0.82, SE = 0.30, 95% CI = 0.29, 1.49), rather than close friends (B = 0.63, SE = 0.31, 95% CI = 0.08, 1.29) [28].

Two studies demonstrate a more complex relationship between frequency of use and disordered eating.

Assessing the use of Instagram in relation to orthorexia nervosa—a disordered eating pattern characterized by an obsession with eating very healthy meals—Villa et al. [30] found that both <1 h/day (OR = 2.77, 95% CI [0.78–9.88]) and >3 h/day (OR = 1.80, 95% CI [0.62–5.26]) were associated with higher odds of ON risk relative to 1–3 h/day. [30]. Similar complexity shows a study by Walker et al. [31]: when Facebook intensity (a measure of Facebook engagement daily) was added to the model with other psychological covariates, it did not predict disordered eating (β = −0.07, *p* = 0.410). However, after accounting for appearance comparison and online fat talk, Facebook intensity became a weak but statistically significant negative predictor of disordered eating (β = −0.12, *p* = 0.046) [31].

Only one study assessed differences between countries, reporting that although a significant positive correlation emerged in Chinese university students (r = 0.29, *p* < 0.01), SM use was unrelated to disordered eating amongst a Japanese sample (r = 0.01, *p* = 0.775). An inconsistent mediation was reported in the study: body esteem mediated the relationship between SM use intensity and ED tendencies for Chinese students wherein higher SM being associated with greater body satisfaction, which in turn was associated with lower ED tendencies (B = −0.08, SE = 0.04, 95% CI [−0.16, −0.02]). Given small effects, platform-specific “intensity” (likely WeChat), and untested measurement invariance, this result should be interpreted with caution [32].

Null results are reported by six studies, with the association between the two variables not reaching significance (all *p* > 0.05) [5,33,34,35,36,37,38]. One study showed that after controlling for depression and the impact of the COVID-19 pandemic, social media use did not predict disordered eating [33]. Similarly, another study showed no difference in disordered eating scores between individuals with mild, moderate, high and excessive TikTok use [38]. We examine the rest of studies in detail in the following sections, highlighting models that adjusted for content type, usage patterns, and other platform-specific factors.

### 3.3. Type of Content and DE

Seven cross-sectional studies assessed the impact of the type of content on DE [5,27,39,40,41,42]. Overall, a significant association emerges between fitness and thinness-related content and disordered eating.

One study showed that exposure to weight loss content was a significant independent predictor of binge eating (BE) frequency (B = 2.54, 95% CI: [1.14–3.94], β = 0.22, *p* < 0.003). Importantly, a significant interaction was observed; time on SM predicted higher binge eating frequency, but only among those exposed to weight loss content (interaction; B = 0.01, 95% CI: [0.01 to 0.02], β = 0.22, *p* < 0.003). Furthermore, neither exposure to body positivity content nor the interaction between exposure to body positivity content and time spent online was found to be a significant predictor of eating outcomes (all *p* > 0.05) in the study [5].

Viewing fitness and diet-related content was associated with drive for thinness (‘Fitspo conten’t: r = 0.236, *p* < 0.01; Clean Eating content: r = 0.374, *p* < 0.01) and bulimia symptoms (Fitspo content: r = 0.127, *p* < 0.05; Clean Eating ‘thinspo’ content: r = 0.233, *p* < 0.01) in a study by Wu et al. [39]. Similar results are reported in another study, showing that exposure to eating-disorder salient content was associated directly with restricting (average direct effect = 0.10, *p* < 0.001, OR = 1.11), with the mediation through negative affect being significant (average causal mediated effect = 0.02, *p* < 0.001, OR = 1.02). No direct or indirect relationship was found between ED-salient content and purging (all *p* > 0.05) [27]. In relation to orthorexia, involvement with health and fitness accounts (‘fitspo’ content) predicted orthorexia both directly (γ = 0.204, *p* < 0.001) and indirectly through thin-ideal internalization and muscular-ideal internalization (all *p* < 0.001) [40].

In an experimental study, both intentional and incidental exposure to fitspiration and thinspiration content significantly increased DE amongst these groups, in comparison to the control condition (F [2, 231] = 6.23, *p* = 0.002, partial η^2^ = 0.051) [42]. However, another study shows mixed results: when participants were shown either a food-focused or a travel-focused Instagram feed, results demonstrated a significant within-subjects interaction between condition and ED behavior intentions in a US Midwestern subsample (F [1, 217] = 323.373, *p* < 0.001), but not for the US Southeastern sample. In the Midwestern sample, participants who were randomly assigned to view the food images reported greater increases in intentions to engage in disordered eating compared to participants who viewed the travel images (partial η^2^ = 0.03). No effects were noted on other variables such as body image or self-esteem (all *p* > 0.05) [41]. It is important to note that experimental research may have limited utility in this context, as it does not capture the cumulative effects of repeated exposure over time.

### 3.4. Type of Social Media Use and DE

Two studies assessed the type of use [39,43]. Given the limited evidence, findings are only presented descriptively.

Examining active and passive use in thinspiration/fitspiration content, Wu et al. [39] found that although individuals who post such content are more likely to engage in compulsive exercise (r = 0.313; *p* < 0.01), there was no association between active use and disordered eating or thin-ideal internalization (all *p* > 0.05). Contrastingly, as aforementioned, passively viewing such content (passive use) was significantly associated with drive for thinness and bulimia symptoms. For viewing fitspiration content, both athletic (indirect effect B = −0.08, 95% CI [−0.14, −0.02], *p* < 0.01) and thin-ideal internalization (indirect effect B = 0.26, 95% CI [0.17, 0.35], *p* < 0.001) significantly mediated the relationship with DE. For active use, the athletic-ideal internalization fully mediated the relationship between posting such content and disordered eating symptoms (indirect effect B = −0.05, 95% CI [−0.10, −0.02], *p* < 0.01). Thin-ideal internalization, however, was not a significant mediator (*p* > 0.05) [39].

Furthermore, in another study, viewing food-related content on social media (passive use) was associated with each of the subscales of the Eating Habits Questionnaire (EHQ) measuring orthorexia, as well as most of the subscales of the Eating Disorder Examination (EDE) (all *p* < 0.05). For sharing such content (active use), correlations with the subscales were weaker, with many not reaching significance. In the same study, through latent profile analyses, three groups were identified with different patterns of SM use. Individuals who had a high risk for orthorexia/DE but did not showcase elevated weight concerns were more likely to both share and view such content in comparison to a normative group. Specifically, their odds of viewing such content were 1.82 times higher than the normative group (OR = 1.82, 95% CI [1.25–2.65], *p* < 0.01), and their odds of sharing were 2.23 times higher (OR = 2.23, 95% CI [1.46–3.41], *p* < 0.001). Yet, individuals who had both DE behaviors and cognitions had significantly higher passive use (with odds 1.60 times higher than the normative group; OR = 1.60, 95% CI [1.13–2.27], *p* < 0.01), without significant differences observed in their active use in relation to the normative group (OR = 1.23, 95% CI [0.81–1.90]). Low-risk individuals showed significantly lower sharing behavior (OR = 0.33, 95% CI [0.12–0.91], *p* < 0.05) with their viewing behavior not differing from normative groups [43].

### 3.5. Other SM Variables and Disordered Eating

Limited research examined variables relating to online behaviors and experiences. These are explored below.

Online self-presentation was found in one study to impact disordered eating. Wick and Keel [44] found that individuals who posted edited pictures of themselves had higher disordered eating scores than those who did not engage in this behavior. Similarly, in Cohen et al. [36] photo investment was linked to greater bulimia symptomatology (β = 0.22, t = 3.25, *p* = 0.001; ΔR^2^ = 0.04) but not drive for thinness (*p* = 0.24). This association was moderated by self-objectification (ΔR^2^ = 0.23, F(6,230) = 6.83, *p* < 0.001), being significant among women high in self-objectification (b = 0.11, t(230) = 2.89, *p* = 0.004) but not at low or average levels.

Investment in online interactions was found to be significantly related to disordered eating in one study. Assessing a SM behavior called social grooming (refers to the creation and display of bonds, support of online relationships, and assertion of hierarchies and alliances online), the study reported significant positive correlations between this behavior and excessive concerns with weight coupled with the pursuit of extreme slimness (drive for thinness) (r = 0.22, *p* = 0.003). However, no associations were found with the preoccupation to become more muscular (drive for muscularity scale). This relationship was fully mediated by appearance comparisons online [37]. Similarly, in another study, although social media variables were not related to disordered eating, reassurance seeking online was. This was measured through the importance one places on receiving feedback or validation from others on Facebook, and was found to be a significant predictor (β = 0.22, t = 6.80, *p* < 0.001, R^2^ = 0.06) [34].

Two studies assessed negative online experiences [35,45]. One longitudinal study reported that when controlling for eating and weight concern at baseline, receiving a higher frequency of negative comments increased both weight and eating concerns at follow-up (b = 0.01, t [147] = 2.10, *p* = 0.04). This relationship was not found for comments that were less negative [45]. Finally, perceived negative social media experience, assessed through a self-report item, was found to be predictive of body surveillance (Instagram β = 0.25, B = 2.23, SE = 0.48, *p* < 0.001; Snapchat β = 0.26, B = 2.24, SE = 0.60, *p* < 0.001, upward comparisons (Instagram β = 0.10, B = 1.20, SE = 0.59, *p* = 0.031; Snapchat β = 0.17; B = 2.23, SE = 0.70, *p* < 0.001) all of which positively correlated with body dissatisfaction which predicted purging, binge eating and restraint (all *p* < 0.001) [35].

### 3.6. COVID-19: Comparison of Studies

During the COVID-19 pandemic, social media served as a tool for social connection, support, and information sharing [33]. As a result, it was important to assess whether differences in the relationship under investigation existed between studies conducted prior to, during, and following the COVID-19 pandemic. Studies were divided into two groups based on whether their publication date was before 2020 (pre-COVID-19) or after 2020 (during and post-COVID-19).

Studies published pre-pandemic demonstrate mixed findings regarding the frequency of social media use [7,25,28,31,34,35,36,37,45]. Three studies reported significant associations, with frequency predicting disordered eating (adjusted OR = 1.68) [7]; drive for thinness correlating with Facebook usage (r = 0.17) [28]; and social media addiction being significantly associated with disordered eating, both directly and indirectly through appearance and weight esteem mediators [25]. However, four studies reported null findings for the direct association between general social media use and disordered eating [34,35,36,37]. Notably, specific behaviors showed associations: negative social media experiences [35], online social grooming behaviors [37], and receiving negative comments [45]. Walker et al. (2015) found that Facebook intensity became a negative predictor of disordered eating (β = −0.12) after accounting for appearance comparison and online fat talk [31].

Similarly, post-pandemic research showed mixed findings [5,24,26,27,29,30,32,33,38,39,40,41,42,43,44]. General social media frequency or duration did not independently predict disordered eating in four adjusted models [5,26,30,33]. Social media addiction was identified as an independent risk factor in one large population study [24], while mixed, cross-cultural studies were found in a different study [32]. One study showed a complex U-shaped relationship, with high and low use predicting disordered eating [30]. Post-pandemic research demonstrated a shift toward content specificity and conditional relationships. Exposure to eating-disorder salient content [27], fitspiration and thinspiration content [5,42], and involvement with health and fitness accounts [40] all predicted disordered eating outcomes. Photo-related behaviors, particularly posting edited photos, were identified as significant predictors [44]. The distinction between active and passive use was studied post-COVID-19, with passive viewing showing stronger and more direct associations with disordered eating, while active posting showed weaker or mediated associations [39,43].

### 3.7. Qualitative Studies

Four studies used semi-structured interviews in order to explore the impact of social media on eating habits and behaviors. All included only female participants [46,47,48,49].

Kaylor et al. (2023) [46] identified a theme labeled as “social media influences and the concept of self-worth” (p. 6) among other themes. Through thematic analysis, they found that participants described social media as an important influence on their eating and exercise behaviors. They observed that social media fosters tendencies to compare oneself to others. Although participants acknowledged that what is displayed online differs from reality, they expressed difficulty recognizing the blurred lines between the digital world and real life [46]. Using an online survey with open-ended questions, Raggatt et al. (2018) [49] found four themes relating to fitspiration content; firstly, they found that all participants reported fitspiration as setting an appearance ideal, which results in the adoption of rigid health goals. Furthermore, failure to achieve these goals results in feelings of inadequacy, a pattern identified as the second theme. Other themes identified showed that a. participants value the sense of community focused on the same goals as them, but b. sometimes leads to being exposed to unqualified advice [49].

Furthermore, in a study by Ando et al. (2021) [47], amongst four themes identified in the study, one was focused on social media. The theme, named “media as a background for interpersonal appearance pressures” (p. 361), emphasized the role of social media in strengthening societal appearance ideals and creating pressure to diet, purchase products, or even engage in cosmetic surgery. Interestingly, this experience differed in more rigid cultural environments, where social media was perceived to be one of the few spaces where young women could explore their preferences and identify in relation to their appearance [47]. Similar complexity is reflected in the study by Hogue et al. (2023) [48], whose research resulted in the identification of 10 themes that the researchers combined into a theory called the ‘dialectical theory of social media and body image’. More specifically, they showed a general pattern of internal conflicts about the impact of social media on participants’ thoughts, emotions, and behaviors. After being shown the thinspiration and fitspiration content, participants were asked how they felt. They reported simultaneously experiencing admiration and jealousy, motivation and discouragement, as well as hope and hopelessness. The researchers explained that the conflict between self-acceptance and the pursuit of societal ideals has resulted in deep internal tensions and contradictions [48].

## 4. Discussion

The current review aimed to present and summarize current knowledge on the association between social media and disordered eating. It focused on studies with individuals between the ages of 18–30 and assessed a variety of social media-related measures, including addiction, frequency, content type, and user behaviors. Furthermore, it included qualitative research of social media users to provide a more in-depth understanding of the experiences of young people.

Overall, findings from this review demonstrate a complex, yet meaningful, relationship between SM use and DE. Such complexity was expected, given that social media environments differ across individuals and depend on their patterns of engagement [50]. Consistent with this view, consumption of fitspiration and thinspiration content emerged as more consistent predictors of disordered eating across research studies. The following sections address each of the associations assessed, with the objective of linking current findings to past literature and shedding light on gaps that should be addressed by future research.

### 4.1. Addiction to Social Media and DE

Prior research shows a significant association between addiction to social media and disordered eating [51,52]. Several theories have been put forth to explain this relationship: the self-presentational theory, according to which individuals who already struggle with their body image are more likely to find the internet appealing [53]; or the emotional coping hypothesis wherein social media use is a coping strategy for individuals with emotion regulation deficits—which is also a transdiagnostic factor in eating disorders [54].

However, we found a limited number of studies assessing these variables for our review (N = 3). Furthermore, our search initially also included adolescent populations, yet limited evidence exists within this group as well (N = 3). From the three studies reported in this review, and three studies with adolescent populations identified [55,56,57], a pattern in support of prior conclusions begins to emerge. It is important, however, to control for potentially confounding factors, such as increased exposure to thinspiration and fitspiration content amongst SM-addicted individuals, in order to further examine this relationship and understand the underlying mechanisms [58]. Although complex and intertwined, separating these social media variables could provide further insights into both the sociocultural and addiction-focused models explored above.

### 4.2. General Social Media Use and DE

A significantly higher number of studies assessed frequency and/or duration of SM use. These demonstrate considerable heterogeneity, limiting our ability to draw conclusions. The tools used, the platforms, and the mediators assessed differed significantly. Collectively, the findings indicate that the relationship between frequency of social media use and disordered eating in young adults is not uniformly positive and is substantially influenced by mediating variables such as anxiety, appearance comparison, thin-ideal internalization, as well as by cultural context and analytical approaches. As a result, it is likely that frequency of use may serve as a distal risk factor whose effects become contingent upon more proximal psychological and social processes.

Significant support for the importance of body dissatisfaction and appearance comparisons as mediating variables emerged. As prior literature supports, social media provides a medium for individuals to compare themselves to others frequently, which results in increased body dissatisfaction and disordered eating behaviors [59,60]. Indeed, in some research where these mediating variables were controlled, a negative association between the two variables emerged, where higher SM use was related to decreased DE, potentially shedding light on some positive features of SM, such as social connection and peer support [29,31]. However, more research needs to be conducted to reach an informed conclusion.

It is also important to note that a higher frequency of use results in more sophisticated algorithmic suggestions. Therefore, it is possible that individuals with predispositions to DE cultivate, through increased use, a digital environment that reinforces one’s maladaptive beliefs and amplifies their intensity [50]. Indeed, the content users are exposed to is arguably one of the most significant drivers of the association between SM use and disordered eating symptomatology. Multiple studies confirm that exposure to weight loss, thinspiration, or appearance-focused content predicts binge eating, drive for thinness, bulimia symptoms, and orthorexia tendencies, both directly and indirectly through mechanisms such as thin and muscular-ideal internalization [5,27,38,39,61]. Researchers underscore the need for young adults to consciously manage the content they engage with on social media, in order to alter their feed suggestions [5].

Finally, an analysis of pre- and post-COVID-19 research demonstrated a shift in research towards content and behavior-specificity, while showing similar patterns in regard to research assessing general use.

### 4.3. Other Social Media Factors

Past research generally indicates that passive social media use is more detrimental to mental health than active use [62]. Consistent with this, the two studies in the present review that directly compared the two usage patterns show stronger associations for disordered eating [38,43]. However, findings on other behaviors point to a more complex interplay between active and passive use: self-presentation online (measured through posting edited images—a behavior that would be categorized as active use) was associated with higher disordered eating scores [36,44]; investment in online interactions was associated with disordered eating [34,37]; negative, online interactions predicted body surveillance and disordered eating behaviors [45]. Although no conclusions can be drawn in relation to these results, they point to the need for future research that investigates how vulnerability factors interact with patterns of use to produce different outcomes for individuals. Special attention could also be paid to online self-presentation behaviors for adolescents as well, as emerging research shows significant associations with such behaviors and DE amongst this population [63,64,65,66].

### 4.4. Qualitative Findings

A rich understanding of the influences of social media on eating behaviors and body image amongst women was explored in qualitative research studies [46,47,48,49]. Across these studies, social media was explored as a tool that simultaneously offers a community and health-related motivation, while cultivating a focus on appearance that can elevate risk for disordered eating. These findings capture the complexity seen in quantitative results: social media can be a tool for connection, but how it is experienced is deeply contextual and contingent on individual differences.

### 4.5. Limitations

Most of the studies included in this review involved cross-sectional designs, limiting the ability to establish causal relationships. In addition, most of the papers were carried out in Western countries, with an undergraduate college population, limiting the ability to generalize findings. The majority of participants in the cross-sectional studies were female, and all participants in qualitative research studies were female. This limits the ability to extend findings to both male and non-binary individuals. However, it reflects the state of research and demonstrates the need to focus more specifically on populations that are underrepresented.

Furthermore, while all scales assess disordered eating, some scales also include maladaptive cognitions in relation to body image and food. For example, the Eating Disorder Examination Questionnaire focuses primarily on behavioral outcomes, while the Eating Attitudes Test-26 assesses both thoughts and behaviors typical of eating disorders. As a result, these may contribute to inconsistencies identified in the findings across studies. Moreover, social media use is mostly assessed through self-report questionnaires. Future research should focus on obtaining objective data from devices, such as time spent on platforms, usage patterns, and online behaviors.

Additionally, the complex interplay of mediating and moderating variables (body dissatisfaction, appearance comparisons, anxiety) makes it difficult to isolate the independent effects of social media use. Studies do not adequately or consistently control for all relevant confounding variables, making it challenging to draw informed conclusions. For example, when assessing passive use, it was not clear whether active use was controlled for.

Similarly, the broad focus of this review on all disordered eating patterns contributed to the heterogeneity in findings. Furthermore, as studies focusing on interventions on social media were not assessed, possible positive effects emerging from these were not considered in this review. Finally, as research in this field is growing at an accelerated pace, it is likely that several studies were published after our search was conducted, as well as that many are in the process of submission. These have not been included in this review.

Future research must adopt more consistent measures, ideally combining subjective self-reports with objective usage data when measuring social media use, to strengthen the comparability and real-world applicability of findings in this field. Longitudinal research assessing a series of individual factors and social media variables could provide further insights into what factors drive maladaptive outcomes.

Finally, more focused research efforts on the content that young adults consume, and how it relates to their eating habits (using objective measures) and attitudes, can aid in the development of targeted preventative interventions. If this association between exposure to weight loss, thinspiration, or eating-disorder salient content and disordered eating persists, public health initiatives should prioritize media literacy that equips young people to critically evaluate this information. Furthermore, teaching young adults how to attend to and recognize signs of weight and appearance concerns, as well as when and how to consciously modify their algorithms to promote healthier online environments, can be beneficial by promoting self-monitoring and early help-seeking.

## 5. Conclusions

The body of research reviewed provides evidence for a complex association between social media use and disordered eating among 18–30-year-olds. Risk appears to be both person-specific (anxiety, internalized ideals) and online context-related (thin and fit-ideal content). Across studies, the content consumed emerged as the most consistently supported risk factor. Future research should focus its efforts on understanding how individual factors interact with the digital environment (e.g., content consumed) to increase disordered eating risk, in order to inform the development of preventative interventions.

## Figures and Tables

**Table 1:** Summary of included research studies.

Author (Date) [Reference]	Method	Main Results
Sanzari et al. (2023) [5]	Survey, in two cohorts (2015, 2022)	The main effect of exposure to weight loss, but not time spent on SM, is on the frequency of binge eating. Interaction effect: more time on SM was associated with BE if exposed to appearance content.
Aparicio-Martinez et al. (2019) [7]	Survey and indirect measure of perinatal testosterone.	In adjusted models, frequency of SM use but not social media addiction was related to disordered eating attitudes.
Karam et al. (2023) [24]	Survey	Addiction to social media was associated with higher odds of emotional overeating. Addiction to SM was more prevalent amongst younger participants.
Murray et al. (2016) [25]	Survey	Significant correlations between excessive social media use, emotional overeating, and restrained eating. Partial mediation through appearance and weight esteem.
Murley et al. (2024) [26]	Survey	Social media predicted food and alcohol disturbance and disordered eating. Indirect paths through anxiety were significant at lower and moderate levels of support, but not at high levels, suggesting a protective mechanism.
Christensen-Pacella et al. (2024) [27]	7-day Ecological Momentary Assessment.	Exposure to DE-salient content was associated with restricting directly, and indirectly, through negative affect. No direct or indirect significant associations were found between DE-content and purging.
Fardouly and Vartanian (2015) [28]	Survey	Drive for thinness was significantly, but weakly, correlated with Facebook usage. Appearance comparisons mediated the association; the effect was stronger for comparisons to distant peers in comparison to close friends.
Foster et al. (2022) [29]	Survey	The frequency of Snapchat use was associated with drive for thinness. Paths for compensatory drinking and eating behaviors were mediated through comparisons and internalization of the thin ideal. When accounting only for appearance comparisons, Snapchat use showed a small but significant negative association with DE.
Villa et al. (2022) [30]	Survey	Spending more than 3 h, as well as less than 1 h in Instagram, was predictive of orthorexia nervosa.
Walker et al. (2015) [31]	Survey	When accounting for other variables, Facebook intensity showed a negative association with disordered eating.
Bai et al. (2024) [32]	Survey	Social media use was associated with disordered eating for Chinese but not Japanese students. Partially mediated by body esteem. Among individuals with good body esteem, social media use was associated with less risk for disordered eating.
Bronfman et al. (2023) [33]	Survey	Social media use was not associated with disordered eating after accounting for depressive symptomatology and the impact of COVID-19.
Howard et al. (2017) [34]	Survey	Increased Facebook use predicted body dissatisfaction but not disordered eating. More reassurance seeking online predicted both more body dissatisfaction and disordered eating. Associations were not moderated by race.
Saunders and Eaton (2018) [35]	Survey	Perceived negative social media experience predicted body surveillance and upward comparisons. These positively correlated with body dissatisfaction, which predicted purging, binge eating, and restraint.
Cohen et al. (2018) [36]	Survey	After accounting for known risk factors, selfie activities online, rather than general social media usage, were associated with eating concerns and bulimia symptomatology. Self-objectification was found to moderate the relationship.
Kim and Chock (2015) [37]	Survey	Social grooming was associated with drive for thinness but not drive for muscularity. Appearance comparisons mediated the former association.
Blackburn and Hogg (2025) [38]	Survey	No difference between low, mild, moderate and extreme TikTok use in relation to disordered eating outcomes as measured by the ORTO-15 and EAT-26.
Wu et al. (2022) [39]	Survey	Fitspiration and clean eating material were significantly positively associated with athletic-ideal internalization. However, only viewing (not posting) fitspiration and clean eating content was significantly related to thin-ideal internalization and disordered eating symptomatology. Athletic-ideal internalization mediated the relationships between active use and DE. Both thin-ideal internalization and athletic-ideal internalization mediated the relationship between passive use and DE.
Scheiber et al. (2023) [40]	Survey	Involvement with health and fitness accounts was associated with orthorexia nervosa. Thin-ideal and muscular internalizations mediated this relationship, but appearance comparison or body dissatisfaction did not.
Kinkel-Ram et al. (2022) [41]	Experiment	Main effect of condition: Watching appearance-focused content in comparison to the control conditions increased DE intentions amongst the US Midwestern site, but not the Southeastern site.
Gracias et al. (2024) [42]	Experiment	Disordered eating was significantly worse in the intentionally exposed group and the incidentally exposed group compared to the unexposed group.
Levin et al. (2023) [43]	Survey	Viewing this thinspiration content was associated with disordered eating. Weaker, but significant associations were found with active use.
Wick et al. (2020) [44]	Experiment	Posting edited photos was associated with increased DE. No differences between the groups were found for depressive symptomatology. Editing photos without posting led to decreases in weight and shape concerns.
Hummel et al. (2015) [45]	Longitudinal study	Individuals with a negative feedback-seeking style who received a high number of comments on Facebook were more likely to report disordered eating attitudes four weeks later.
Kaylor et al. (2023) [46]	Qualitative interviews.	Social media impacts eating habits and fosters self-comparison. Blurred lines between reality and Instagram impact self-esteem.
Ando et al. (2021) [47]	Qualitative interviews.	The media acts as a background for interpersonal appearance pressures.
Hogue et al. (2023) [48]	Qualitative interviews	Young women understand both the positive effects of social media, such as social support and a means for self-expression, but expressing concern with the impact it has on self-perception and eating behaviors.
Raggat et al. (2018) [49]	Qualitative interviews.	Participants reported both positive (accessing information, being part of a community) and negative (setting a healthy ideal, failing to meet the ideal) influences of appearance-focused content.

## Abbreviations

The following abbreviations are used in this manuscript:

SM	Social Media
DE	Disordered Eating
FAD	Food and Alcohol Disturbance
ED	Eating Disorder
EAT-26	Eating Attitudes Test-26
EDE-Q	Eating Disorder Examination Questionnaire
SCOFF	Sick, Control, One stone, Fat, Food scale
EHQ	Eating Habits Questionnaire
ORTO-15/ORTO-11	Orthorexia Scales (15-item; 11-item)
BE	Binge Eating
CI	Confidence Interval
ME	Mediated Effect
SE	Standard Error
